# Adaptive Diffeomorphic Multiresolution Demons and Their Application to Same Modality Medical Image Registration with Large Deformation

**DOI:** 10.1155/2018/7314612

**Published:** 2018-05-16

**Authors:** Chang Wang, Qiongqiong Ren, Xin Qin, Yi Yu

**Affiliations:** ^1^School of Biomedical Engineering, Xinxiang Medical University, Xinxiang 453003, China; ^2^Key Lab of Neurosense and Control, Xinxiang Medical University, Xinxiang, Henan 453003, China

## Abstract

Diffeomorphic demons can guarantee smooth and reversible deformation and avoid unreasonable deformation. However, the number of iterations needs to be set manually, and this greatly influences the registration result. In order to solve this problem, we proposed adaptive diffeomorphic multiresolution demons in this paper. We used an optimized framework with nonrigid registration and diffeomorphism strategy, designed a similarity energy function based on grey value, and stopped iterations adaptively. This method was tested by synthetic image and same modality medical image. Large deformation was simulated by rotational distortion and extrusion transform, medical image registration with large deformation was performed, and quantitative analyses were conducted using the registration evaluation indexes, and the influence of different driving forces and parameters on the registration result was analyzed. The registration results of same modality medical images were compared with those obtained using active demons, additive demons, and diffeomorphic demons. Quantitative analyses showed that the proposed method's normalized cross-correlation coefficient and structural similarity were the highest and mean square error was the lowest. Medical image registration with large deformation could be performed successfully; evaluation indexes remained stable with an increase in deformation strength. The proposed method is effective and robust, and it can be applied to nonrigid registration of same modality medical images with large deformation.

## 1. Introduction

Nonrigid registration has been applied to intersubjective registration to detect lesions and establish atlas. Comparisons of nonrigid registration algorithms have shown that those with demons based on optical flow field theory are superior [1].

The demons algorithm was initially only applicable to image registration with small deformation. Therefore, many studies have tried to improve it. In 2005, Wang et al. introduced the floating image gradient in the diffusion equation and proposed active demons [2, 3]. In 2006, Rogelj and Kovačič proposed symmetric demons algorithm and demonstrated that it is the most efficient [4]. In 2007, Vercauteren et al. applied an optimized framework with nonrigid registration to demons and proposed additive demons. In their method, nonrigid registration was equivalent to the optimization of similarity energy function, and iterations could be stopped adaptively [5]. In 2008, symmetric log-domain diffeomorphic demons algorithm was proposed, and log Euclidean was used in order to avoid time-consuming computations of log spatial transformations [6]. In 2009, diffeomorphic demons algorithm was proposed and it was shown that the final deformation is topologically invariant and that unreasonable deformations are not produced [7]. In 2010, Xu et al. introduced a regularization term and designed a new similarity energy function to ensure smooth and reversible deformations [8]. In 2012, Lei et al. proposed new active gradient and curvature demons (G&C) using the curvature to control the deformation [9].

In recent years, some studies have proposed nonrigid image registration methods with large deformation. Lombaert et al. introduced the direct feature matching technique to find global correspondences between images and the proposed spectral log-demons registration with large deformation [10, 11]. In 2015, Zhao and Jia used multilayer convolutional neural networks to determine scale and translation parameters and proposed the deep adaptive log-demons method for diffeomorphic image registration with very large deformations [12]. In 2015, Yan et al. performed image registration with large deformation by combining manifold learning and diffeomorphic demons [13].

In this paper, we proposed an adaptive diffeomorphic multiresolution demons algorithm and used an optimized framework with nonrigid registration and the diffeomorphic deformation strategy. First, a similarity energy function based on the grey value was designed as a registration metric, and a termination condition was set based on the variation of this metric. Iterations could be stopped adaptively, and medical image registration with large deformation was performed. This method was tested by synthetic image and same modality medical image with large deformation simulated by rotational distortion and extrusion. Mean square error, normalized cross-correlation, and structural similarity were used as evaluation indexes to verify the superiority of this method. Medical image registration with large deformation simulated by rotational distortion and extrusion was performed successfully. Quantitative analyses of the influences of different driving forces and parameters on the registration result showed that our method is effective and robust. This method can be applied to same modality medical image registration with large deformation.

## 2. Materials and Methods

### 2.1. Additive Demons

Nonrigid image registration is essentially a multiparameter optimization problem involving mapping a moving image to a reference image. It defines an appropriate objective function as the registration metric and then optimizes this metric. For reference image *F* and moving image *M*, the registration metric should be optimized to seek the best transform *t*^opt^. *t* : Rn → Rn, *p* → *t*(*p*) is the mapping of pixel *p* of the moving image to pixel *t*(*p*) of the reference image. The definition of the registration metric is a crucial step in the nonrigid registration process. The mean square deviation based on the grey value as a registration metric can be calculated using

(1) Sim⁡F,M∘t=12F−M∘t2=12ΩP∑P∈ΩPFp−Mtp.

In (1), *Ω* is the common region of *F* and *M* after registration and ∘ is the transform operator. Directly minimizing the registration metric using (1) will lead to an unstable solution, and therefore a regularization term needs to be added to restrict the geometric transform. The revised energy function *E*(*t*) is expressed using

(2) Et=1σiSim⁡F,M∘t+1σtRe⁡gt.

In (2), *σ*_*i*_ is the local noise level and *σ*_*t*_ is the regularization parameter. Cachier et al. introduced the parameters *c* (nonruled spatial transform) and *t* (ruled spatial transform). Then, the new energy function is expressed using

(3) Ec,t=1σiF−M∘c2+1σx2Distc,t2+1σtRe⁡gt.

In (3), Dist(*c*, *t*) = ‖*c* − *t*‖ and *σ*_*x*_ is the uncertainty degree between *c* and *t*. The displacement field *u* is produced using the space geometric transform, and two vectors are added directly to form a new vector. Vercauteren et al. proposed additive demons, and the energy function comprised a similarity measure term, deformation error term, and regularization term. The final energy function is expressed as follows:

(4) $$\begin{array}{c} E\left(\;u\;\right)=\frac{1}{{\sigma_{i}}^{2}}{\left\|\;F-M\circ \left(\;t+u\;\right)\;\right\|}^{2}+\frac{1}{{\sigma_{x}}^{2}}{\left\|\;u\;\right\|}^{2}. \end{array}$$

In (4), *u* is the updated displacement field and Dist(*c*, *t*) = ‖*c* − *t*‖ = ‖*u*‖. By minimizing the energy function and solving the displacement field, the final displacement field is expressed using

(5) $$\begin{array}{c} u\left(\;p\;\right)=-\frac{F\left(p\right)-M\circ t\left(p\right)}{{J_{P}}^{2}+{\sigma_{i}}^{2}\left(p\right)/{\sigma_{x}}^{2}}{J_{P}}^{T}. \end{array}$$

In (5), *σ*_*i*_(*p*) = |*F*(*p*) − *M*∘*t*(*p*)| and *J*_*P*_ = −∇^*T*^*F*(*p*).

### 2.2. Diffeomorphic Deformation Strategy

To ensure that the deformation is smooth, reversible, and topologically invariant, a diffeomorphic space was proposed. Diffeomorphic transform, which is based on the theory of Lie groups, is related to the exponential map of the velocity field *v*; that is, *ϕ* = exp⁡(*v*), and a practical and fast approximation method with a scaling-and-squaring strategy was described as shown in Algorithm 1.

**Figure 1:**
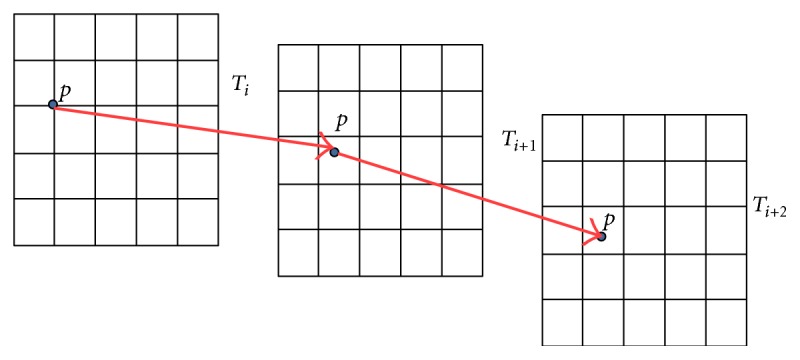
Superposition of deformation field.

In the process of registration, deformation field formation is based on the superposition principle, and it is shown in Figure 1.

### 2.3. Algorithm Implementation

The diffeomorphic deformation strategy was used for optimizing the energy function designed by additive demons. The deformation should always be topologically invariant. Rough deformation greatly influences the registration result; if it is calculated unsuitably, it easily falls into a local optimum. Therefore, a multiresolution strategy was used, and rough deformation was calculated with a low resolution. Then, the energy function was minimized and the best registration result was obtained by avoiding the local optimum.

The number of iterations also greatly influences the registration result. If the number of iterations is insufficient, the best deformation field cannot be obtained. The time consumed will increase with the number of iterations. The mean square deviation based on the grey value was designed as a registration metric, and iterations could be stopped adaptively depending on the variation of the energy function. This can eliminate the influence of the number of iterations on the registration result. The convergence condition was defined by

(6) if En−En−1<En−1∗stop_criterium breakelse continue.

When updating the displacement field, three different demons driving forces were proposed as follows: Thirion's primitive driving force, *J*_*P*_ = −∇^*T*^*F*(*p*); Gauss-Newton-based advanced driving force, *J*_*P*_ = −∇^*T*^(*M*∘*t*(*p*)); and symmetric driving force, *J*_*P*_ = −(∇^*T*^*F* + ∇^*T*^(*M*∘*t*(*p*)))/2.

**Figure 2:**
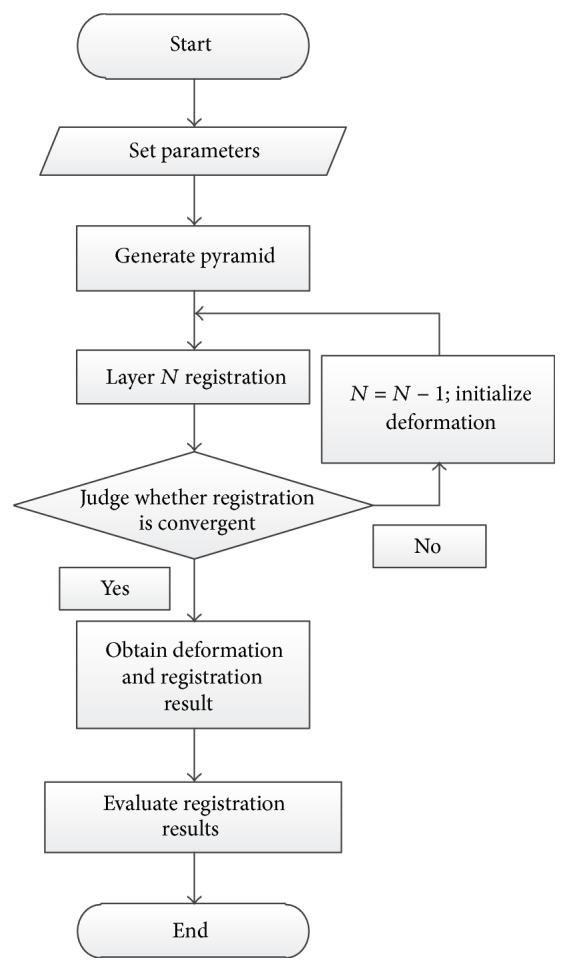
Algorithm flow.

Figure 2 shows the algorithm flow.

The steps for each registration layer can be described as follows.

**Step 1 .**

Initialize displacement field.

**Step 2 .**

Calculate demons driving force *u*(*p*) and update velocity field *v*.

**Step 3 .**

Regulate deformation field using Gauss filter.

**Step 4 .**

Obtain exponential mapping of deformation field by diffeomorphic transform.

**Step 5 .**

Calculate similarity measurement function *E*(*t*) using (4).

**Step 6 .**

Judge convergence condition.

**Step 7 .**

Transform the moving image by the diffeomorphic deformation field.

**Step 8 .**

Interpolate the moved image by nearest neighbor interpolation algorithm.

### 2.4. Algorithm Evaluation

Mean square error, normalized cross-correlation, and structural similarity [14] were calculated as evaluation indexes. The mean square error can be defined by

(7) $$\begin{array}{c} \text{MSE}=\sqrt{\frac{\sum {\left(S\left(x,y\right)-M\left(x,y\right)\right)}^{2}}{n}}. \end{array}$$

The normalized cross-correlation coefficient was written by

(8) $$\begin{array}{c} \text{CC}=\frac{\sum \left(S\left(x,y\right)-\bar{S}\right)\left(M\left(x,y\right)-\bar{M}\right)}{\sqrt{\sum {\left(S\left(x,y\right)-\bar{S}\right)}^{2}}\sqrt{\sum {\left(M\left(x,y\right)-\bar{M}\right)}^{2}}}, \end{array}$$

where *S* is the reference image, *M* is the registration result, and $\bar{S}$ and $\bar{M}$ are the average grey values of each pixel point in the reference image and registration result, respectively.

## 3. Results

The superiority of our method is verified through comparisons with active demons, additive demons, and diffeomorphic demons. The experimental parameters were set as follows: Gaussian filter parameter *G*_*σ*_, where *σ* = 2; updating step of deformation field *σ*_*x*_ = 1.0; multiresolution layer number *N* = 3; and convergence condition stop criterion = 0.005.

### 3.1. Synthetic Image Registration

**Figure 3:**
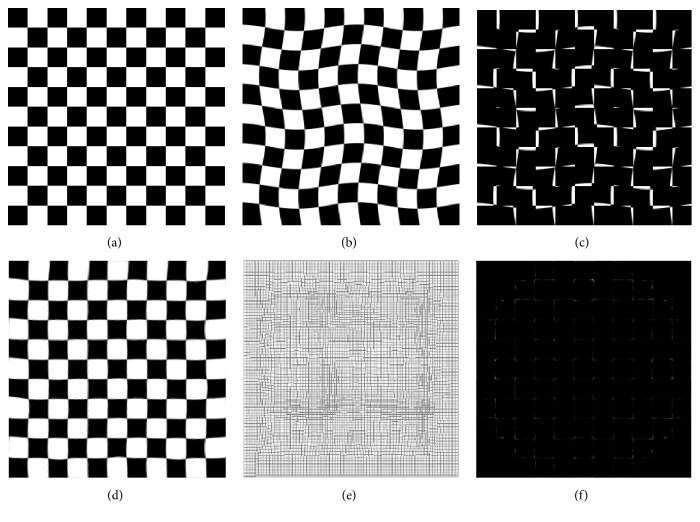
Results of checkboard image. (a) Reference image, (b) moving image, (c) initial difference, (d) registration result, (e) deformation, and (f) difference after registration.

The checkboard image as a synthetic image was processed to validate our method, and the result is shown in Figure 3.  Figure 3(a) is the reference image, Figure 3(b) is the moving image, Figure 3(c) is the difference between reference and moving images, Figure 3(d) is the registration result, Figure 3(e) is the final deformation, and Figure 3(f) is the difference between reference and registration results. It can be seen that this method can realize nonrigid image registration and estimate deformation field effectively.

### 3.2. Same Modality Medical Image Registration

**Figure 4:**
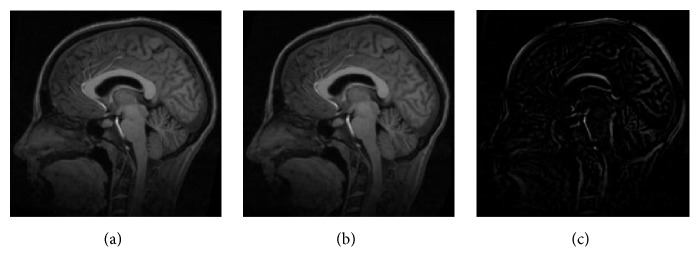
Medical image. (a) Reference image, (b) moving image, and (c) initial difference.

Medical image was selected from Simulated Brain Database provided by Montreal Neurological Institute, McGill University. The size of the medical image is 217 × 181 × 181, and T1-weighted MR images were selected as reference and moving images. The intensity scale of origin medical images and registration result was converted to [0~255]. Figures 4(a) and 4(b) are reference and moving images, and Figure 4(c) is the initial difference between reference and moving images.

**Figure 5:**
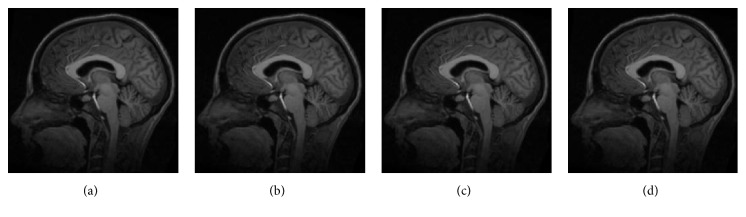
Registration result. (a) Our method, (b) diffeomorphic demons, (c) additive demons, and (d) active demons.

**Figure 6:**
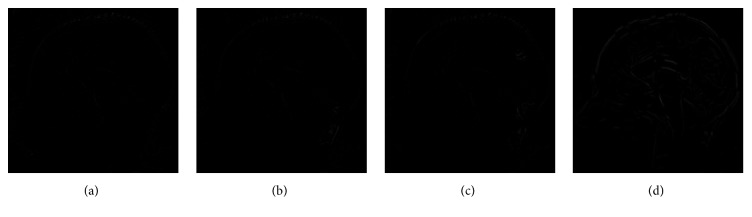
Difference between registration result and reference image. (a) Our method, (b) diffeomorphic demons, (c) additive demons, and (d) active demons.

**Figure 7:**
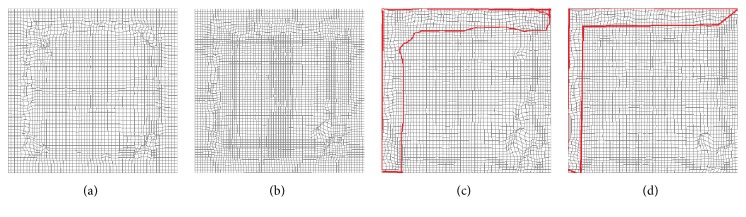
Deformation field. (a) Our method, (b) diffeomorphic demons, (c) additive demons, and (d) active demons.

Medical image registration was completed by active demons, additive demons, diffeomorphic demons, and our method, and the result is shown in Figures 5–7.  Figure 5 is the registration result by different methods, Figure 6 is the difference between registration result and reference images, and Figure 7 is the final deformation field. From Figures 5–7, it can be seen that our method can obtain the best registration result and topological invariant deformation field. The edge of deformation field is more reasonable than active demons and additive demons.

Table 1 shows the quantitative analysis of the evaluation indexes for MRI image registration. It is seen that the normalized cross-correlation coefficient and structural similarity are the highest and the mean square error is the lowest; therefore, our method is superior to active demons, additive demons, and diffeomorphic demons.

### 3.3. Medical Image Registration with Large Deformation

Large deformation was produced by two types of free transforms: rotational distortion and extrusion. The deformation field was determined by the transform strength.

**Figure 8:**
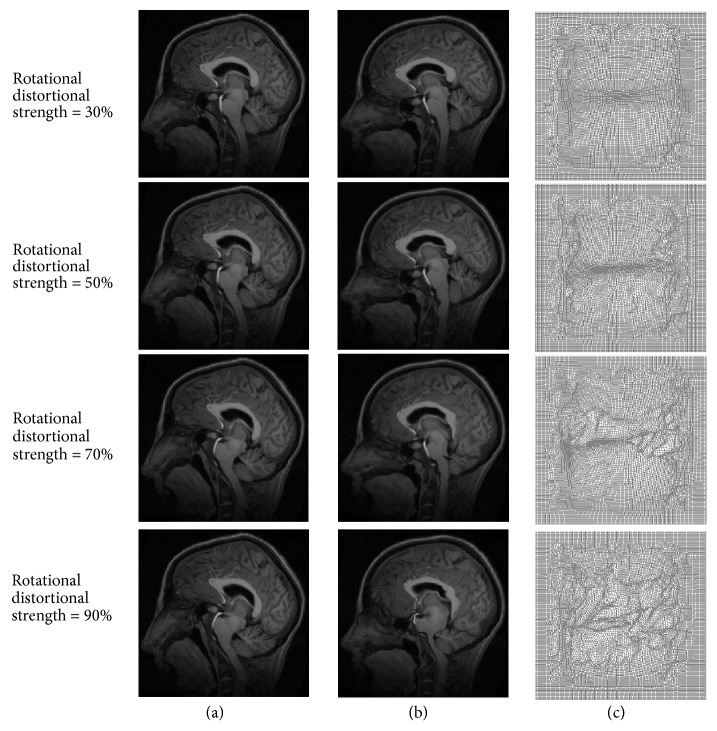
Medical image registration with large deformation produced by rotational distortion. (a) Moving images produced for rotational distortional strengths of 30%–90%, (b) corresponding registration results, and (c) corresponding final deformation fields.

Medical image registration with large deformation produced by rotational distortion was performed using our proposed method, as shown in Figure 8. Figure 8(a) shows moving images produced with rotational distortional strengths of 30%–90%, Figure 8(b) shows the corresponding registration result, and Figure 8(c) shows the corresponding final deformation field.  Table 2 shows the quantitative analysis result; the normalized cross-correlation coefficient and structural similarity decreased and the mean square error and time consumed gradually increased with an increase in the deformation field strength.

**Figure 9:**
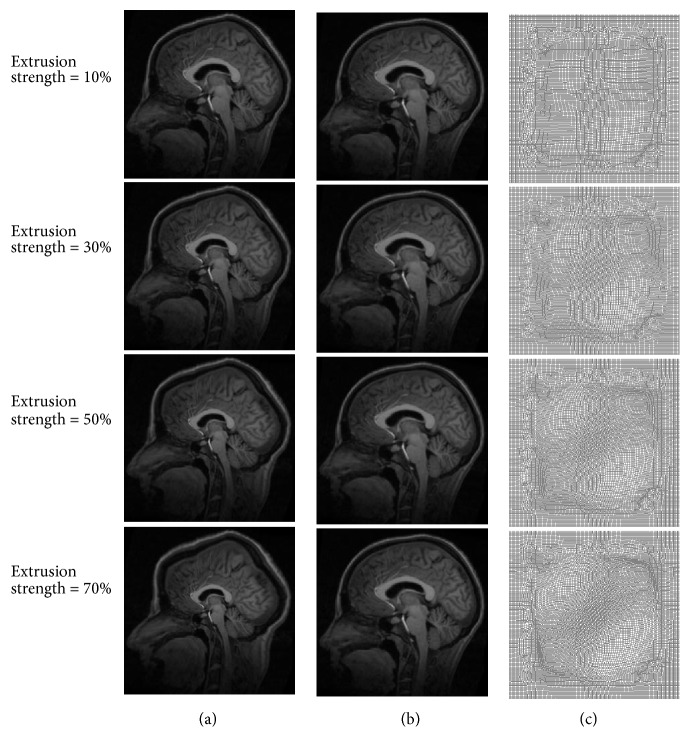
Medical image registration with large deformation produced by extrusion. (a) Moving images produced for extrusion strengths of 10%–70%, (b) corresponding registration results, and (c) corresponding final deformation fields.

Figure 9 shows the result for large deformation produced by extrusion. Figure 9(a) shows moving images produced for extrusion strengths of 10%–70%, Figure 9(b) shows the corresponding registration result, and Figure 9(c) shows the corresponding final deformation field.  Table 3 shows the quantitative analysis result; the normalized cross-correlation coefficient, structural similarity, and mean square error basically remained stable and the time consumed gradually increased with an increase in the deformation field strength. Therefore, medical image registration with large deformation produced by both rotational distortion and extrusion can be performed efficiently.

### 3.4. Analysis of Different Driving Forces

**Figure 10:**
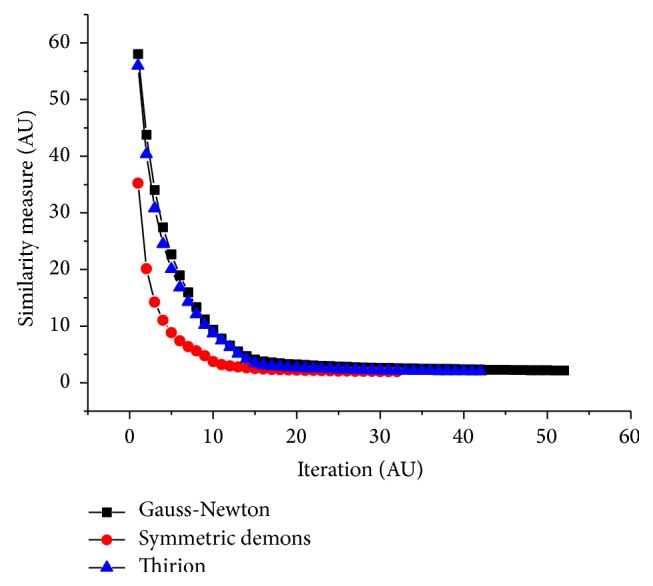
Comparison of three different demons driving forces.

Thirion, Gauss-Newton, and symmetric driving forces were used, and the displacement field was defined differently.  Figure 10 shows the variation of the energy function *E*(*t*); the convergence is the fastest and the energy function of driving forces is the lowest with symmetric driving forces.  Table 4 shows the quantitative analysis; the symmetric driving force shows the best performance.

### 3.5. Influence of Parameters on Registration Result

The Gaussian filter, deformation updating step length, resolution layer, and convergence condition were used as experimental parameters. The resolution layer can only influence the registration speed, and the convergence condition can influence the registration accuracy.  Table 5 shows details about the deformation updating step length; the registration accuracy remained stable and the time consumed decreased with an increase in the step length from 0.8 to 2.0.

## 4. Conclusion

This study proposed adaptive diffeomorphic multiresolution demons for medical image registration with large deformation. An optimized framework with nonrigid registration and the diffeomorphism strategy was used, a similarity energy function based on grey value was designed, and termination conditions were set to stop iterations adaptively. This method was applied to synthetic image and same modality medical image registration, and mean square error, normalized cross-correlation, and structural similarity were used as evaluation indexes to verify its superiority. Medical images with large deformation were simulated by rotational distortion and extrusion transform, and quantitative analyses showed that they could be registered successfully and efficiently using our method. The influence of different driving forces shows that our method based on symmetric driving force is the most efficient, and the influence of parameters on the registration result shows that our method is robust. This method can solve same modality medical image registration problem with large deformation and can be applied clinically.

## Figures and Tables

**Algorithm 1:**
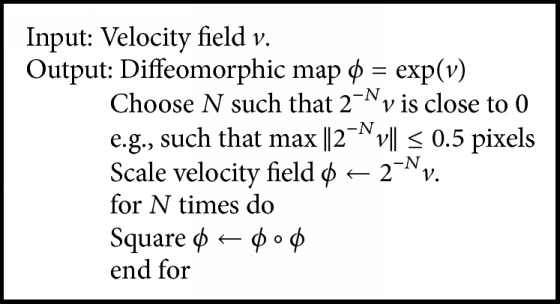
Exponential *ϕ* = exp⁡(*v*).

**Table 1:** Evaluation of registration result.

Experiment method	MSE (AU)	NCC (AU)	Structural similarity (AU)
Our method	514.7965	0.9993	0.9952
Diffeomorphic demons	583.0147	0.9992	0.9937
Additive demons	640.9294	0.9987	0.9906
Active demons	1307.5	0.9944	0.9824

**Table 2:** Evaluation of registration result (rotational distortion deformation).

Rotational distortional strength	MSE (AU)	NCC (AU)	Structural similarity (AU)
30%	531.6939	0.9991	0.9950
50%	1763.9	0.9898	0.9479
70%	2948.6	0.9717	0.9107
90%	5046.0	0.9169	0.8136

**Table 3:** Evaluation of registration result (extrusion deformation).

Extrusion strength	MSE (AU)	NCC (AU)	Structural similarity (AU)
10%	543.2	0.9991	0.9940
30%	545.1	0.9991	0.9944
50%	529.8	0.9991	0.9949
70%	559.4	0.9990	0.9946

**Table 4:** Evaluation of registration result based on different demons driving forces.

Experiment method	MSE (AU)	NCC (AU)	Structural similarity (AU)
Thirion	514.79	0.9993	0.9948
Gauss-Newton	476.1	0.9993	0.9948
Symmetric demons	465.0	0.9994	0.9952

**Table 5:** Influence of parameter *σ*_*x*_ on registration accuracy.

Registration accuracy	*σ*_*x*_				
	2.0	1.5	1.2	1.0	0.8
MSE (AU)	464.81	479.2040	485.8915	508.2908	522.31
NCC (AU)	0.9993	0.9993	0.9992	0.9992	0.9991
Structural similarity (AU)	0.9953	0.9949	0.9949	0.9943	0.9939
Time consumed (s)	136.46	150.37	193.94	222.06	287.1
